# Characteristic of HBV nucleic acid amplification testing yields from blood donors in China

**DOI:** 10.1186/s12879-021-06468-y

**Published:** 2021-07-30

**Authors:** Danxiao Wu, Xiaojuan Wang, Fangjun Feng, Dairong Wang, Yiqin Hu, Yang Yu, Jihong Huang, Min Wang, Jie Dong, Yaling Wu, Hong Zhu, Faming Zhu

**Affiliations:** 1 Blood Center of Zhejiang Province, Hangzhou, 310052 Zhejiang People’s Republic of China; 2 Key Laboratory of Blood Safety Research of Zhejiang Province, Jianye Road 789, Hangzhou, 310052 Zhejiang People’s Republic of China

**Keywords:** Nucleic acid amplification test, Blood screening, Windows period, Occult HBV infections, Non-discriminating reactive

## Abstract

**Background:**

Nucleic acid amplification testing (NAT) for blood screening has been previously performed in some countries to determine NAT yields. The current study sought to explore the non-discriminating reactive NAT yields using individual-NAT (ID-NAT) and characteristics of HBV NAT yields through a 10-year retrospective analysis in Zhejiang, China.

**Methods:**

Blood donations were analyzed using individual-NAT mode by the transcription-mediated amplification (TMA) method. Supplementary HBV serological tests were performed using chemiluminescent immunoassay, and HBV viral load assay was performed by real-time polymerase chain reaction. Follow-up studies were performed in partial donors with low HBV viral loads.

**Results:**

Non-discriminating reactive NAT yields and HBV NAT yields varied in different years. The yields ranged from 853.73 per million to 2018.68 per million and 624.60 per million to 1669.50 per million, respectively. In the 476 NAT yields, 19 were probable window periods (WP), 33 probable occult hepatitis B virus infections (OBIs), 409 were confirmed OBIs and 15 were chronic HBV infections. ID-NAT results were categorized in four groups, and the findings showed that the levels of HBV DNA viral loads were different in the four different groups (χ^2^ = 275.02, p < 0.01). HBV viral load distribution was significantly different between anti-HBs positive and anti-HBc positive samples (χ^2^ = 49.429, p < 0.01). Notably, only 42.03% donors were NAT repeated positive in the 138 repeat donors’ follow up tests.

**Conclusion:**

NAT screening of blood donations can reduce the risk of transfusion-transmitted HBV infections. Positive proportions of anti-HBs and anti-HBc are correlated with the HBV viral load level. However, low level of viral load donors pose risks in HBV NAT assays, and show fluctuating state for HBV viral load and leads to non-repeated NAT results during follow up studies.

## Background

Hepatitis B virus (HBV) infection is a major public health concern in the world. The global hepatitis report by World Health Organization (WHO) in 2017 shows that approximately 3.5% of the world’s population has chronic HBV infection. Further, approximately 786,000 people die every year from liver failure, cirrhosis and primary liver cancer caused by HBV infection [1]. Currently, HBV infection is the tenth leading cause of death [1]. High prevalence of HBV infection has been reported in China, and about one-third of global HBV carriers are residents of China. In the past three decades, Chinese government has made great efforts to improve prevention and control of hepatitis B. As a result, the positive rate of HBV surface antigen (HBsAg) decreased from approximately 9.75% in 1992 to less than 3.00% in 2014 [2, 3]. However, approximately 80 million people are HBsAg positive owing to the large population of China.

Vertical transmission, sexual transmission and blood transmission are common routes for spread of HBV infection. Currently, blood donations from HBsAg-negative donors during the pre-seroconversion window period (WP) or OBI stage are defined as an absence of detectable HBsAg in circulation. However, presence of HBV DNA in blood or liver tissue is a major risk of transfusion transmission infections (TTIs) [4]. To decrease the risk of HBV transfusion transmission, infectious markers of the HBV infection should be screened in blood donors including HBsAg and HBV DNA. Nucleic acid amplification testing (NAT) implementation for blood safety was began in 2010 in China. The guideline for virological screening for blood safety is that HIV, HBV and HCV infection markers should be tested twice with serological method in China before December 2015, and the NAT implementation was chosen and not mandatory. From 2016, the control strategy is modified and tested once with NAT and once with serological method by the government, but now most services are still used the strategy for testing twice for serological method and once NAT. Currently, HBV DNA detection in blood donations uses the NAT assay with individual mode (ID-NAT) or mini pool mode (MP-NAT). Studies report that HBV NAT yields rate of occult HBV infection (OBI) carriers ranges between 1:1000 and 1:20,000 [5, 6]. However, some studies report donors who are reactive in the initial multiplex assay but are non-reactive in the discriminatory assays pose challenges in the ID-NAT mode. Thus, may indicate possible OBI infection due to low or fluctuating levels of HBV DNA in blood [7, 8]. Routine NAT screening for blood donations was started in the Blood Center of Zhejiang province, China from August 1, 2010. Notably, several HBV NAT yields donations have been identified through these routine tests thus improving the safety of blood donations. In the current study, HBV NAT yields in donors and non-discriminating reactive yields were explored using ID-NAT mode through a 10-year retrospective analysis in the Blood Center of Zhejiang province, China. Additional serological, viral load tests and follow-up study were performed to further explore the infection status in these donations.

## Methods

### Blood samples

Blood samples used in this study were obtained from voluntary donors attending the Blood Center of Zhejiang province, China from August 1, 2010 to December 31, 2019. Informed consent was obtained from all donors. Donors filled a pre-donation risk factor questionnaire, and underwent pre-examination and per-screening tests including haemoglobin level, HBsAg, ALT level and ABO blood group. After meeting these requirements, the donors donated blood components following the blood donation guideline in China. After donations, blood samples were screened for ALT level and ABO blood grouping. In addition, HBsAg, anti-HCV, anti-HIV and anti-TP were determined using two different enzyme-linked immunosorbent assay (ELISA)commercial kits following the manufacturer’s instructions. Reagents for HBsAg detection were obtained from InTec Products, Xiamen, China and BioMérieux Clinical Diagnostics, Shanghai, China.

### Nucleic acid amplification testing (NAT) assays

Different commercial systems based on transcription-mediated amplification (TMA) method were used for NAT detection from August 1, 2010 to December 31, 2019 following the manufacturer's instructions (Table 1). Analysis included the screening assay and discriminatory assay (Novartis Diagnostics, Emeryville, CA, USA) with ID-NAT mode. If the samples were initially reactive in the screening assay, the final result for the sample was referred as reactive and results in the further repeated tests were disregarded in the screening assay and discriminatory assay.

**Table 1:** Reagents used for screening donors in different NAT systems

System		Procleix® Tigris® system		Procleix® Panther® system
Methods		TMA, individual NAT for screening and discriminatory assays		
Time range for using		2010.8.1–2015.7.31	2015.8.1–2016.9.21	2016.9.22–2019.12.31
Kit name (Company)		Procleix® Ultrio® Assay (Novartis Diagnostics, Emeryville, CA, USA)	Procleix® Ultrio Plus® Assay (Novartis Diagnostics, Emeryville, CA, USA)	Procleix® Ultrio Elite® Assay (Novartis Diagnostics, Emeryville, CA, USA)
Sensitivity (IU/mL, 95%LOD)	HBV (ID-NAT)	10.4 (9.2–12.2)	3.4 (3.0–4.1)	4.3 (3.8–5.0)
	dHBV	8.5 (7.6–9.8)	4.1 (3.5–4.9)	4.5 (4.0–5.3)

Analysis of samples with initial reactive was performed two times using the same screening assay and two times through discriminatory assay. Therefore, the samples were categorized in four groups based on the results of repeated tests and discriminatory assay. (1) non-repeated positive group was defined as repeat screening NAT assay and discriminatory NAT assay for non-reactive HBV DNA, HCV RNA, and HIV-1 RNA; (2) non-discriminated positive group was defined as repeat screening NAT assay reactive but discriminatory NAT assay non-reactive for HBV DNA, HCV RNA, and HIV-1 RNA; (3) non-repeated HBV-DNA positive group was defined as repeat screening NAT assay reactive and discriminatory NAT assay for HBV DNA was once reactive but could not be repeated; (4) repeated HBV-DNA positive group was defined as repeat screening NAT assay reactive and discriminatory NAT assay for HBV DNA was reactive on repeating.

### Supplementary assays

Further serological tests for NAT yields samples included HBsAg, antibody to HBsAg (anti-HBs), hepatitis B E antigen (HBeAg), antibody to HBeAg (anti-HBe), and antibody to hepatitis B core antigen (anti-HBc). These tests were performed by electrochemiluminescence immunoassay (ECLIA) using a Cobas e601 analyzer (Roche Diagnostics Company, Shanghai, China) or chemiluminescent immunoassay (CLIA) with an ARCHITECTTM i2000SR analyzer (Abbott Diagnostics, Abbott Park, IL). In addition, HBV NAT yield cases were tested for viral load using the Roche Cobas AmpliPrep with real-time polymerase chain reaction performed on a Cobas TaqMan analyzer (Roche Diagnostics Company, Shanghai, China). Notably, the manufacturer states that the lower limit of detection for HBV DNA assay is 12 IU/mL. Some samples were detected using alternative NAT using TaqMan PCR method (Roche Diagnostics Company, Shanghai, China). All analysis procedures were performed following the manufacturer’s instructions.

### Follow-up study

Repeat blood donors whose HBV viral load test was negative or below 12 IU/mL were followed up for more than two weeks interval until loss to follow-up. Follow-up samples were analyzed for HBV serological markers and HBV DNA.

### Statistical analysis

SPSS 22.0 statistical software was used for statistical analyses. Differences among various groups or years were analyzed using the chi-square test. A p < 0.05 was considered statistically significant.

## Results

### Total HBV NAT yields in blood donations

**Fig. 1:**
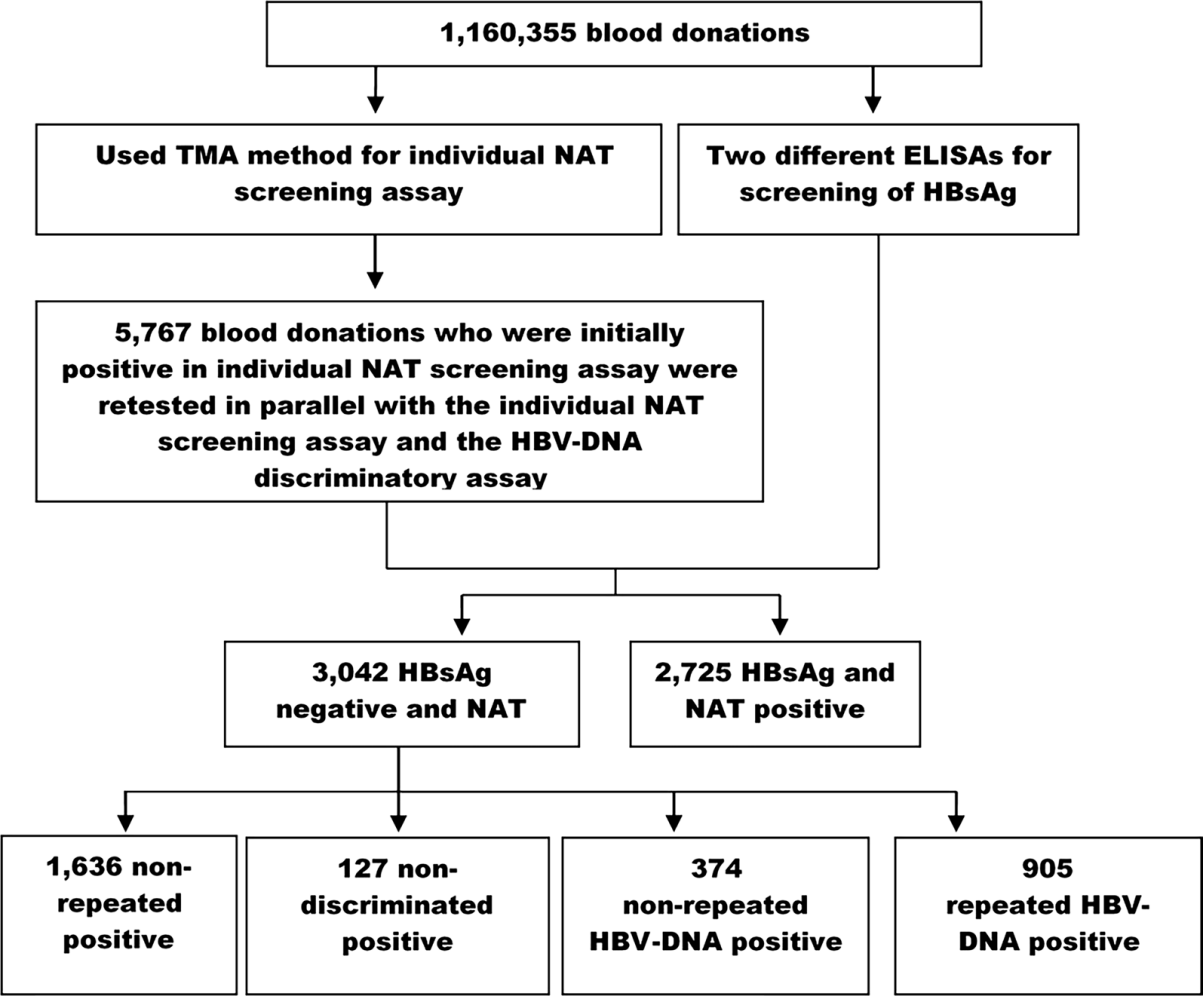
Flow chart for ID-NAT analysis

A total of 1,160,355 blood donations were analyzed by ID-NAT from August 1, 2010 to December 31, 2019. Notably, 3042 NAT yields donations were obtained based on the initial reactive results with exception of two HCV and three HIV NAT yields. Out of the 3042 NAT yields, 1279 donations were verified as confirmatory HBV NAT yields cases, including 374 non-repeated HBV-DNA positive donations and 905 HBV-DNA positive donations. In addition, 1763 blood donations were HBsAg negative and NAT positive but were discriminated as non-reactive, including 1636 non-repeated positive and 127 non-discriminated positive donations. A flow chart of this study is showed in Fig. 1. The donations were categorized in four distinct groups using the TMA method.

### NAT yield rates of the four groups varied in different years

Three assays were used in this study, including Procleix® Ultrio® assay, Procleix® Ultrio Plus® assay and Procleix® Ultrio Elite® assay (Table 1). NAT yield rates of the four groups were different in each year. Analysis of the rate in each group was significantly different among different years (Table 2, χ^2^ = 81.888, p < 0.01 in non-repeated positive; χ^2^ = 53.953, p < 0.01 in non-discriminated positive; χ^2^ = 64.626, p < 0.01 in non-repeated HBV-DNA positive; χ2 = 118.531, p < 0.01 in repeated HBV-DNA positive). Furthermore, analysis showed significant differences in HBV NAT yields (including non-repeated HBV-DNA positive and repeated HBV-DNA positive groups) and non-discriminating reactive rate (including non-repeated positive and non-discriminated positive groups) across the different years (Table 2, χ^2^ = 150.546, p < 0.01 in HBV NAT yields; χ^2^ = 55.122, p < 0.01 in non-discriminating reactive).

**Table 2:** The yield rates of the four groups were varied in the different years

HBV NAT yields			Years										Total
			**2010**	**2011**	**2012**	**2013**	**2014**	**2015**	**2016**	**2017**	**2018**	**2019**	
Types	Donations Groups	Number	46,853	131,594	125,656	105,668	119,301	106,505	106,494	158,791	136,568	122,925	1,160,355
Non-discriminating reactive	Non-repeated positive	Number	31	123	162	144	168	209	180	243	228	148	1636
		NAT yields (per million)	661.64	934.69	1289.23	1362.76	1408.20	1962.35	1690.24	1530.31	1669.50	1203.99	1409.91
	Non-discriminated positive	Number	9	31	27	13	14	6	6	8	7	6	127
		NAT yields (per million)	192.09	235.57	214.87	123.03	117.35	56.34	56.34	50.38	51.26	48.81	109.45
	Total	Number	40	154	189	157	182	215	186	251	235	154	1763
		Proportion	51.28%	60.63%	61.76%	70.40%	66.67%	69.13%	63.48%	50.30%	50.76%	45.03%	57.96%
		NAT yields (per million)	853.73	1170.27	1504.11	1485.79	1525.55	2018.68	1746.58	1580.69	1720.75	1252.80	1519.36
HBV	Non-repeated HBV-DNA positive	Number	13	35	42	32	23	15	15	78	73	48	374
		NAT yields (per million)	277.46	265.97	334.25	302.84	192.79	140.84	140.85	491.21	534.53	390.48	322.32
	Repeated HBV-DNA positive	Number	25	65	75	34	68	81	92	170	155	140	905
		NAT yields (per million)	533.58	493.94	596.87	321.76	569.99	760.53	863.90	1070.59	1134.97	1138.91	779.93
	Total	Number	38	100	117	66	91	96	107	248	228	188	1279
		Proportion	48.72%	39.37%	38.24%	29.60%	33.33%	30.87%	36.52%	49.70%	49.24%	54.97%	42.04%
		NAT yields (per million)	811.05	759.91	931.11	624.60	762.78	901.37	1004.75	1561.80	1669.50	1529.39	1102.25
Overall		Number	78	254	306	223	273	311	293	499	463	342	3042
		NAT yields (per million)	1664.78	1930.18	2435.22	2110.38	2288.33	2920.05	2751.33	3142.50	3390.25	2782.18	2621.61

### HBV viral load analysis in partial NAT yields cases

A total of 476 NAT yields of blood donations were collected from two successive periods, with 317 donations from January 1, 2011 to February 29, 2012 and 159 donations from January 1, 2017 to February 28, 2018. Out of the 476 NAT yields, 225 were from the non-repeated positive group, 49 were from the non-discriminated positive group, 87 were from the non-repeated HBV-DNA positive group and 115 from the repeat HBV-DNA positive group.

HBV quantitative experiment assay showed that a total of 212 samples were positive. Frequency of HBV quantitative positive and levels of HBV DNA viral loads were significantly different in the four different groups (χ^2^ = 275.02, p < 0.01). Analysis of the non-repeated positive group showed that 78.22% samples were negative and 21.78% were HBV viral loads below 12 IU/ml. Analysis of the non-discriminated positive group showed that 57.14% were negative, whereas 28.57% and 14.29% were HBV viral loads below 12 IU/ml and 12–100 IU/ml, respectively. Analysis of the non-repeated HBV-DNA positive group showed that 48.28% were negative, whereas 24.14% and 27.59% were HBV viral loads below 12 IU/ml and 12–100 IU/ml, respectively. The findings of the repeated HBV-DNA positive group showed that 6.96% were negative, whereas 18.26% and 46.96% and 27.83% were HBV viral loads below 12 IU/ml, 12–100 IU/ml and > 100 IU/ml, respectively.

### Classification of HBV infection

A total of 476 samples with HBV viral load were tested for HBV serological markers (Table 3). The samples were classified into 4 categories based on the HBV viral load and serological markers (Table 3). The groups included 19 (3.99%) samples with probable window period (WP), 33 (6.93%) with probable OBIs, 409 (85.92%) with confirmed OBIs and 15 (3.15%) with chronic HBV infection.

**Table 3:** The classification of HBV NAT yields according to alternative HBV NAT and serological markers

Classification	Groups of HBV NAT yields	Number	Alt NAT#	HBV-DNA (VLs)	HBsAg*	Anti-HBs	Anti-HBc
Probable WP (n = 19)	Non-repeated positive (n = 8)	5	−	−	−	−	−
		3	+	−	−	−	−
	Non-discriminated positive (n = 1)	1	+	−	−	−	−
	Non-repeated HBV-DNA positive (n = 2)	1	+	−	−	−	−
		1	+	+	−	−	−
	Repeat HBV-DNA positive (n = 8)	8	+	+	−	−	−
Probable OBI (n = 33)	Non-repeated positive (n = 28)	28	−	−	−	+	−
	Non-discriminated positive (n = 2)	2	−	−	−	+	−
	Non-repeated HBV-DNA positive (n = 3)	3	−	−	−	+	−
Confirm OBI (n = 409)	Non-repeated positive (n = 187)	5	+	+	−	+	−
		59	+	−	−	−	+
		80	+	−	−	+	+
		20	+	+	−	−	+
		23	+	+	−	+	+
	Non-discriminated positive (n = 45)	1	+	+	−	+	−
		11	+	−	−	−	+
		14	+	−	−	+	+
		13	+	+	−	−	+
		6	+	+	−	+	+
	Non-repeated HBV-DNA positive (n = 75)	1	+	+	−	+	−
		19	+	−	−	−	+
		19	+	−	−	+	+
		23	+	+	−	−	+
		13	+	+	−	+	+
	Repeat HBV-DNA positive (n = 102)	4	+	−	−	−	+
		4	+	−	−	+	+
		80	+	+	−	−	+
		14	+	+	−	+	+
Chronic infection (n = 15)	Non-repeated positive (n = 2)	1	+	−	+	−	+
		1	+	+	+	−	+
	Non-discriminated positive (n = 1)	1	+	+	+	−	+
	Non-repeated HBV-DNA positive (n = 7)	1^&^	+	+	+	−	+
		6	+	+	+	−	+
	Repeat HBV-DNA positive (n = 5)	5	+	+	+	−	+

^#^Alternative NAT by using TaqMan PCR method; * supplemental HBsAg test using CLIA method; “ + ” means a positive result; “-” means a negative result; &, the donation was HBeAg positive; VLs, viral loads; OBI, occult hepatitis B virus infection; WP, window-period infection

Out of the 19 samples negative for all HBV serological markers, 14 samples tested positive by alternate NAT (TaqMan PCR, Roche). Therefore, all samples were defined to have probable WP HBV infection. 409 samples were classified as confirmed OBI based on alternative positive HBV NAT analysis with anti-HBc and/or anti-HBs positive. However, 33 cases were positive with anti-HBs and tested negative using alternative HBV NAT and supplemental HBsAg test. These cases were classified as probable OBI with anti-HBs only. Notably, the HBsAg result in 15 donations showed a weak positive using CLIA test and positive using alternative NAT analysis (Table 3), thus they were classified as low-level chronic HBV carriers.

### Positive proportions of anti-HBs and anti-HBc were correlated with HBV viral load level

Proportions of anti-HBs and anti-HBc were significantly different in the four groups (Table 3). Anti-HBs positive was highest (136/225, 60.44%) in the non-repeated positive group, and lowest (18/115, 15.65%) in the repeated HBV-DNA positive group (χ^2^ = 26.725, p < 0.01). A total of 33 samples (82.50%) tested negative based on viral load analysis of 40 samples with anti-HBs positive only. However, analysis showed that 87.61% samples (417/476) were anti-HBc positive. Anti-HBc positive rate was lowest in the non-repeated positive group (184/225, 81.77%), higher in the non-repeated HBV-DNA positive group (81/87, 93.10%) and the repeated HBV-DNA positive group (107/115, 93.04%).

**Fig. 2:**
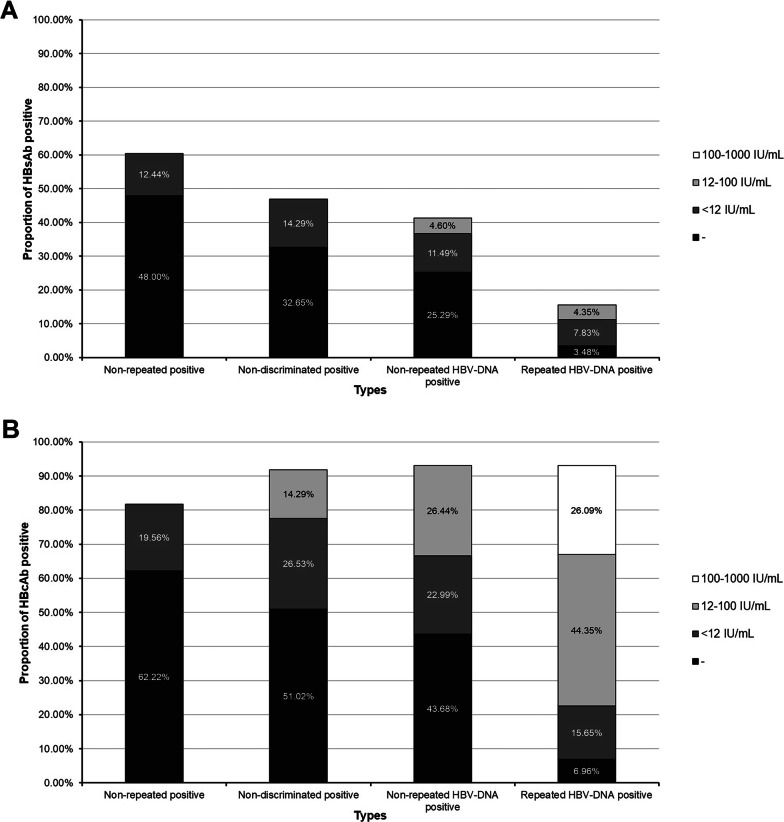
Proportions of anti-HBs (A) and anti-HBc (B) in four different groups. Classification based on concentration of viral loads, showed that samples were negative (■), below 12 IU/mL (■), 12 to 100 IU/mL (■) and 100 to 1000 IU/mL (□)

HBV viral load distribution was significantly different between the anti-HBs positive and anti-HBc positive samples (χ^2^ = 49.429, p < 0.01). This implies that HBV viral loads were correlated with the positive proportions of anti-HBs and anti-HBc. Further classification based on the results of anti-HBs and anti-HBc, showed significant difference in the HBV viral load distribution between the four groups (Fig. 2, χ^2^ = 52.117, p < 0.01).

### Donors with low HBV viral load could be not repeated during follow up study

A total of 138 repeat donors whose viral loads were negative or below 12 IU/mL were followed up till December 31, 2019 (Table 4). Analysis using TMA method showed that only 58 donors (42.03%) were repeated positive in the follow up specimens. Repeated positive rates of the non-repeated positive, non-discriminated positive, non-repeated HBV-DNA positive, repeated HBV-DNA positive groups were 34.62%, 50.00%, 71.43% and 75.00% respectively. Notably, the differences among the groups were not significant (χ^2^ = 3.993, p > 0.05). However, the interval times (months) for the follow-up study were different in these groups, which in the non-repeated positive group was significantly longer compared with that in the other groups, mainly the repeated HBV-DNA positive group (p < 0.05).

**Table 4:** The results of NAT in some repeat donors during the follow up study

Groups of the results in NAT^+^ELISA^−^ donors (numbers)	Non-repeated positive	Non-discriminated positive	Non-repeated HBV-DNA positive	Repeated HBV-DNA positive	Overall
All follow up donors	104	12	14	8	138
NAT^+^ donors in follow up study	36	6	10 (2)*	6	58 (2)
Groups of the results in follow-up study					
Non-repeated positive	18	1	3	1	23
Non-discriminated positive	5	0	0	1	6
Non-repeated HBV-DNA positive	6	2	2 (1)	0	10 (1)
Repeated HBV-DNA positive	7	3	5 (1)	4	19 (1)
The interval time (months) of the NAT^+^ results (± SD)	32.07 (± 27.21)	29.38 (± 23.89)	24.54 (± 19.84)	12.68 (± 13.56)^#^	28.83 (± 25.62)

^*^ The number in () means the donors were in the window period of acute HBV infection. ^#^p < 0.05 compared to the non-repeated positive group

### HBV viral load level fluctuated during follow up

**Fig. 3:**
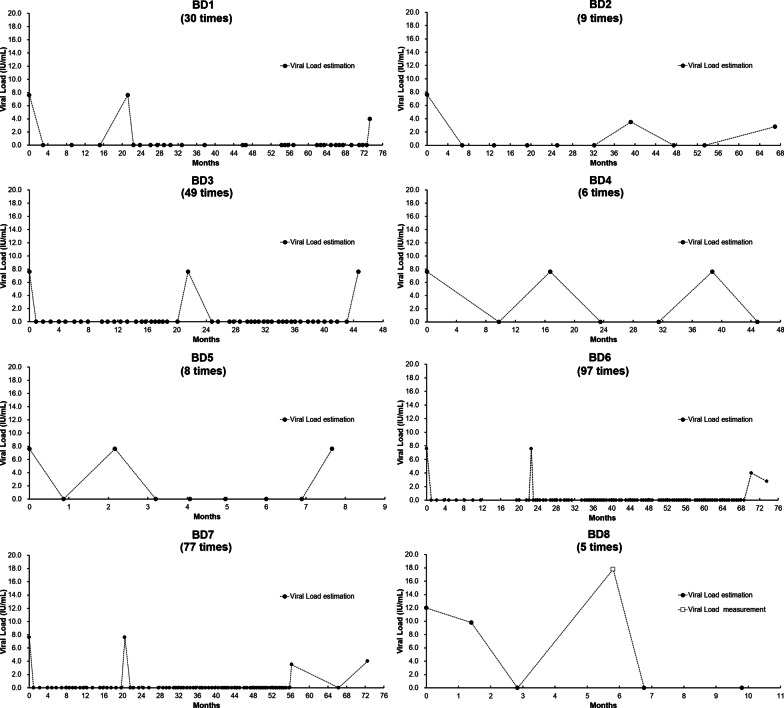
Viral load distribution of 8 donors who were repeat positive in the follow-up study. Viral load was estimated by probit analysis on replicate NAT results (●) or determined by Roche Cobas TaqMan assay (□)

A total of eight OBI donors were detected several times during follow up, including seven non-repeated positive donors (BD1-BD7) and one HBV-DNA positive donor (BD8) (Table 5). The cumulative numbers of the follow-up samples of each non-repeated positive donor were more than six times, and the highest one was 97 times, however, only 3 to 4 times were NAT positive (Fig. 3). LOD of TMA assay (Table 1) showed that the HBV viral loads of these donors were below 7.6 IU/mL (95% LOD in dHBV of Ultrio assays) and showed fluctuating level of HBV viral loads (Fig. 3, BD1-BD7). One donor in the HBV-DNA positive group (BD8) showed higher viral load level compared with those in the non-repeated positive donors. In addition, this donor showed fluctuating state for viral load from negative to 17.8 IU/mL (Fig. 3, BD8).

**Table 5:** The results of repeat donors with low level viral load who were repeat positive in the follow up stud

Donors	Donation date*		Donation type	Age	Gender	Times of Follow-up	NAT result groups	Method for NAT test	Alt NAT#	HBV-DNA (VLs, IU/mL)	Serological marker positive
BD1	X	October 5, 2011	PLT	49 (1970)	Female	30	Non-repeated positive	ULTRIO	/	Neg	Anti-HBc
	X + 21	July 2, 2013	FS				Non-repeated positive	ULTRIO	/		
	X + 73	October 12, 2017	FS				Non-repeated positive	ELITE	/		
	X + 80	May 4, 2018	FS				Neg	ELITE	Pos		
BD2	X	December 10, 2012	PLT	45 (1974)	Female	9	Non-repeated positive	ULTRIO	/	Neg	Anti-HBc
	X + 39	March 2, 2016	FS				Non-repeated positive	PLUS	/		
	X + 67	June 11, 2018	FS				Neg	ELITE	Pos		
BD3	X	April 19, 2011	PLT	62 (1957)	Male	49	Non-repeated positive	ULTRIO	/	Neg	Anti-HBc and anti-HBs
	X + 3	July 16, 2011	FS				Non-repeated positive	ULTRIO	/		
	X + 21	January 27, 2013	FS				Non-repeated positive	ULTRIO	/		
	X + 44	December 20, 2014	FS				Non-repeated positive	ULTRIO	/		
BD4	X	June 3, 2011	WB	55 (1964)	Male	6	Non-repeated positive	ULTRIO	/	Neg	Anti-HBc and anti-HBs
	X + 16	October 16, 2012	FS				Non-repeated positive	ULTRIO	/		
	X + 38	August 9, 2014	FS				Non-repeated positive	ULTRIO	/		
BD5	X	September 28, 2011	PLT	41 (1978)	Male	8	Non-repeated positive	ULTRIO	/	Neg	Anti-HBc and anti-HBs
	X + 1	December 2, 2011	FS				Non-repeated positive	ULTRIO	/		
	X + 7	May 15, 2012	FS				Non-repeated positive	ULTRIO	/		
BD6	X	May 19, 2011	PLT	52 (1967)	Male	97	Non-repeated positive	ULTRIO	/	Neg	Anti-HBc and anti-HBe
	X + 23	March 26, 2013	FS				Non-repeated positive	ULTRIO	/		
	X + 70	February 22, 2017	FS				Non-repeated positive	ELITE	/		
	X + 73	June 1, 2017	FS				Neg	ELITE	Pos		
BD7	X	November 20, 2011	PLT	51 (1968)	Female	77	Non-repeated positive	ULTRIO	/	Neg	Anti-HBc, anti-HBs and anti-HBe
	X + 21	July 25, 2013	FS				Non-repeated positive	ULTRIO	/		
	X + 56	July 3, 2016	FS				Non-repeated positive	PLUS	/		
	X + 72	November 2, 2017	FS				Non-repeated positive	ELITE	/		
BD8	X	September 18, 2010	PLT	43 (1976)	Male	5	HBV-DNA positive	ULTRIO	/	< 12	Anti-HBc
	X + 1	October 30, 2010	FS				HBV-DNA positive	ULTRIO	/	Neg	
	X + 6	March 11, 2011	FS				HBV-DNA positive	ULTRIO	/	17.8	

^*^Months from index donation; #alternative NAT by using TaqMan PCR method, Roche

VLs, viral loads; BD, blood donor; WB, whole blood; PLT, platelet; FS, follow-up sample; /, not applicable; Neg, negative; Pos, positive; ULTRIO, Procleix® Ultrio® assay; PLUS, Procleix® Ultrio Plus® assay; ELITE, Procleix® Ultrio Elite® Assay

## Discussion

The positive rate of HBsAg among residents of Zhejiang province, China was 11.61% in 1990s[9]. Incidence of hepatitis B infection in Zhejiang province has decreased from 93.67/100,000 in 2004 to 24.80/100,000 in 2019 due to adoption of various prevention measures [9, 10]. However, the HBV infection remains a major threat to public health and affects blood safety during donation in Zhejiang province, China. A pilot study was conducted to screen donations by NAT test in blood services of China from 2010, including the Blood Center of Zhejiang province. The current study explored NAT yield in blood screening using ID-mode with 10 years retrospective analysis. Analysis showed that the distribution of HBV NAT yields varied across different years (lowest in 2013 with 624.60 per million and highest in 2018 with 1669.50 per million), which can be attributed to the different screening assays and donors. NAT results were classified into four groups based on the specificity of the ID-NAT mode. In addition to HBV NAT^+^ discriminate results, analysis showed a total of 1763 non-discriminating NAT results. However, some of the non-discriminating NAT results tested positive for HBV DNA using alternative methods or during the follow up period, which was similar with previous findings [8]. In the current study, NAT yields of non-discriminating reactive results showed an overall average of 1519.36 cases per million (0.15%), which was higher compared with findings reported in Korea (0.05%) [11] and New Zealand (0.09%) [8]. However, they were lower compared with that of Shenzhen, China (0.21%) [12] where HBsAg prevalence is higher compared with that of the Zhejiang Province [10]. These findings show that NAT yields of non-discriminating reactive results may be dependent on prevalence of HBV infection, screening assay reagents and strategies used in different studies.

In addition, HBV virus load distribution varied in the NAT non-discriminating reactive results, and was lower compared with that in the HBV-DNA positive group. Notably, the anti-HBs and anti-HBc reactive rates were correlated with HBV viral load level. In the non-discriminating reactive donors, 83.57% (229/274) of cases were anti-HBc positive, which was higher compared with those reported in Korean (47%) [11] and New Zealand (13%-57%) [8] donors and lower compared with that in Shenzhen, China (91.1%) [12]. A significantly large variance was observed for the positive proportions of anti-HBs and anti-HBc, and viral load level in the different groups. Analysis showed that the non-repeated positive donations had low anti-HBc and high anti-HBs positive proportions, and low viral loads in the current study. High anti-HBc and low anti-HBs positive proportions, and high viral loads were observed in the repeated HBV-DNA positive group. Therefore, these findings indicate that anti-HBc and anti-HBs positive rates are correlated with HBV viral loads level. HBV DNA was detected in some anti-HBs positive samples in this study, implying that absence of HBsAg and presence of anti-HBs do not guarantee safety of blood donations. Previous studies reported acute liver failure in immunosuppressed patients [13, 14] after transfusion with blood of OBI cases with anti-HBs. Allain et al. [15] reported that presence of anti-HBs (titer: 20–160 IU/L) in donors reduces the risk of HBV infection by approximately five-fold. Cases of HBV hepatitis have been reported as a result of transfusion of anti-HBc positive, anti-HBs positive (12 IU/L) and HBV DNA positive (180 IU/mL) blood from one donor [16]. However, cases carrying anti-HBc alone are more infectious compared with those with low level of anti-HBs [17].

HBV WP and OBI cases pose major challenges in blood screening. Detection frequency of HBV WP and OBI is directly dependent on sensitivity of assays for either or both HBV markers and HBV prevalence in the populations [18]. The main advantage of NAT screening is interdiction of HBV WP infections and identification of OBI carrier status, offering a significantly higher sensitivity for detecting blood-borne infections [19]. In China, HBV NAT yield rates ranges from 1:1000 to 1:10,000 out of which approximately 20% are pre-seroconversion HBV WP and 80% are OBI [20, 21]. However, in the current study, only 2.94% cases were HBV WP and over 3.15% were chronic HBV infection in the HBV NAT yields. Notably, the ratio of HBV WP cases was lower compared with that reported in a previous study [22], which may be attributed with different donors. All chronic HBV infection cases tested HBsAg negative using ELISA method, however, these cases tested positive using the CLIA test. These findings indicate that the levels of HBsAg in these donations were below the ELISA detection sensitivity level, and the NAT assay was able to detect HBV-DNA much earlier and at low levels compared with ELISA. Therefore, NAT assay can be used to reduce transfusion transmitted HBV infection. Studies have not fully explored whether blood components from OBI donors with low viral DNA levels are infectious, however, the blood donors are asymptomatic and the donations with low HBV levels are intermittently or not detectable, and most of them may become repeat donors. Transfusion of their blood components to patients with weakened immune capacity or immunosuppressive therapy significantly increases the risk of HBV infection caused by blood transfusion, therefore, the risk of HBV TTI from these donors is higher. High proportion of anti-HBc was observed in OBI cases (402/442, 90.95%), which is consistent with findings from previous studies [12, 23]. Studies report that anti-HBc is an important indicator for serological status of HBV infection to exclude false positive results in NAT, mainly in non-repeated positive donors [12, 24, 25].

Several factors such as viral dose, blood component fresh frozen plasma and platelet concentrates suspended in plasma (considered more infectious compared with red cell concentrates), presence of anti-HBs, and the recipient immune status affect infectivity of HBV [15]. Notably, the neutralizing capacity of low anti-HBs may be ineffective when overcome by high viral load [26]. Satake et al. [27] reported that blood components obtained from OBI donors with low levels of anti-HBc are more than tenfold less infectious compared with units collected from donors with HBV WP of infection. Candotti et al. [28] reported HBV transmissions by blood components from three repeat donors who tested negative for HBsAg and HBV DNA with a highly sensitive screening test (limit of detection 3.4 IU/mL). In the follow-up analysis of the low viral load OBI donors in the current study, 57.97% negative results were reported using NAT assays, implying that the viral load in these donors were near or below the detection limit of the assay. However, some repeat donors were analyzed several times during follow up and had fluctuating level of HBV viral load, which showed positive or negative NAT results in the follow up samples, indicating that these blood donations may have HBV infectivity. Therefore, based on blood safety as reported in this study and previous studies [23, 26–28], even if the sample was initially reactive in the screening assay using ID-NAT, the blood of this donor should be discarded and not used for transfusion.

In summary, NAT screening in blood donations can detect HBV WP and OBI donations, and reduce risk of transfusion-transmitted HBV infections. In the current study, HBV yield rates varied and were dependent on sensitivity of the screening assay and HBV prevalence of the blood donors. However, low level of viral load cases is a threat to residual risks in HBV NAT assays, which may cause missed detection in NAT screening mainly the discriminate assay or in follow up samples. The viral load level in these cases exhibit fluctuating state, which can affect the blood safety.

## Data Availability

All data used in this study is available from the corresponding author upon reasonable request.

## Abbreviations

ALT: Alanine aminotransferase
Anti-HBc: Antibody to hepatitis B core antigen
Anti-HBe: Antibody to hepatitis B E antigen
Anti-HBs: Antibody to hepatitis B surface antigen
CLIA: Chemiluminescent immunoassay
dHBV: Discriminatory test for HBV DNA
DNA: Deoxyribonucleic acid
ECLIA: Electrochemiluminescence immunoassay
ELISA: Enzyme linked immunosorbent assay
HBeAg: Hepatitis B E antigen
HBsAg: Hepatitis B virus surface antigen
HBV: Hepatitis B virus
HCV: Hepatitis C virus
HIV: Human immunodeficiency virus
ID: Individual donation
LOD: Limit of detection
MP: Mini pool
NAT: Nucleic acid amplification testing
OBI: Occult hepatitis B virus infection
PCR: Polymerase chain reaction
RNA: Ribonucleic acid
TMA: Transcription-mediated amplification
TTIs: Transfusion transmission infections
WP: Window period
